# Development of Infrared Prediction Models for Diffusible and Micellar Minerals in Bovine Milk

**DOI:** 10.3390/ani9070430

**Published:** 2019-07-09

**Authors:** Marco Franzoi, Giovanni Niero, Mauro Penasa, Massimo De Marchi

**Affiliations:** Department of Agronomy, Food, Natural resources, Animals and Environment, University of Padova, Viale dell’Università 16, 35020 Legnaro (PD), Italy

**Keywords:** mineral, micellar, diffusible, milk, spectroscopy, infrared

## Abstract

**Simple Summary:**

Minerals are distributed in milk in two main forms: The diffusible (or soluble) fraction, composed of free ions and inorganic salts, and the micellar fraction, composed of mineral elements located on the surface or in the inner part of casein micelles. The ratio between diffusible and micellar minerals strongly affects milk coagulation ability. The objective of this study was to investigate the ability of mid-infrared spectroscopy to predict diffusible and micellar Ca, P, K, Mg and Na in individual milk samples of Holstein Friesian cows. Overall, the accuracy of mid-infrared prediction models was moderate for Ca, P and Mg, and low for micellar K, micellar Na and diffusible Na.

**Abstract:**

Milk and dairy products are major sources of minerals in human diet. Minerals influence milk technological properties; in particular, micellar and diffusible minerals differentially influence rennet clotting time, curd firmness and curd formation rate. The aim of the present study was to investigate the ability of mid-infrared spectroscopy to predict the content of micellar and diffusible mineral fractions in bovine milk. Spectra of reference milk samples (n = 93) were collected using Milkoscan™ 7 (Foss Electric A/S, Hillerød, Denmark) and total, diffusible and micellar content of minerals were quantified using inductively coupled plasma optical emission spectrometry. Backward interval partial least squares algorithm was applied to exclude uninformative spectral regions and build prediction models for total, diffusible and micellar minerals content. Results showed that backward interval partial least squares analysis improved the predictive ability of the models for the studied traits compared with traditional partial least squares approach. Overall, the predictive ability of mid-infrared prediction models was moderate to low, with a ratio of performance to deviation in cross-validation that ranged from 1.15 for micellar K to 2.73 for total P.

## 1. Introduction

Milk and dairy products are important sources of minerals for human health, with particular regard to physiological processes such as cellular homeostasis, bone growth, muscular and nervous functions and blood clotting. On average, dairy products provide 59% and 27% of recommended daily intake of Ca and P, respectively, and 10% of K and Mg [1]. From a technological point of view, milk minerals are directly involved in casein micelles stability, the milk coagulation process and cheese yield [2,3]. In particular, during the cheese-making process, minerals are differentially partitioned into curd and whey, and the relative proportion of these two fractions is influenced by milk composition and technological treatments [4]. Minerals associated to curd, named micellar minerals, can be incorporated into casein micelles as colloids, or nanoclusters. Still, some micellar minerals associate to casein micelles, even if they are not part of colloids; for example, Ca is present on the micellar surface, forming molecular bridges responsible for paracaseinate complex formation [5]. The remaining amount of minerals is defined as diffusible, and may be present in the form of free ions or associated with counter ions and proteins [6,7,8]. Significant sources of variation for milk minerals are management, lactation stage and parity, whereas the seasonal effect is trivial [9,10].

Several methods have been proposed to discriminate between diffusible and micellar mineral fractions. Rennet coagulation-, ultracentrifugation-, dialysis- and ultrafiltration-based methods are widely used, and their efficiency has been already discussed [11]. Recently, Franzoi et al. (2018) [12] proposed a modification of the rennet coagulation-based method to overcome the need of using correction factors for the excluded volume. Indeed, correction factors have been usually applied to all the samples, notwithstanding the single sample variation in major constituents and quality [13]. Once the fractionation of micellar and diffusible milk minerals is achieved, the quantitation is usually carried out through inductively coupled plasma optical emission spectrometry after mineralisation using microwave-assisted acid digestion [14]. Regardless of the adopted methods, sample preparation and quantification of minerals are expensive, time-consuming and require specific instrumentation and skilled operators.

Mid-infrared spectroscopy (MIRS) is a rapid, non-destructive and cost-effective method for the determination of milk components, routinely used in dairy companies [15,16]. Several studies reported a moderate ability of MIRS to predict milk mineral composition and, even if accuracies do not suggest the use of this technique for milk payment or analytical purposes, it can be useful for population studies [17,18,19,20,21]. Variable selection procedures have shown to be useful tools to improve the accuracy of MIRS prediction models [17]. Among them, backward interval partial least squares (BiPLS) algorithm has shown good performance and effectiveness in external validation procedures [22,23]. To date, only Malacarne et al. (2018) [17] have investigated the ability of MIRS to predict micellar and diffusible minerals, but only in bulk milk samples. Those authors collected reference data using the ultrafiltration procedure to differentiate between micellar and diffusible minerals, demonstrating the poor performance of MIRS prediction models, likely attributable to the low variability of the studied traits [17]. Therefore, the aim of the present study was to develop MIRS prediction models for total, micellar and diffusible minerals of individual cow milk, by applying BiPLS as the variable selection and prediction algorithm.

## 2. Materials and Methods

### 2.1. Chemical Analysis

A total of 93 Holstein cows (days in milk = 168.66 ± 115.27; parity = 2.78 ± 1.61; milk yield = 32.08 ± 8.49 kg/day) were sampled during morning milking between April and June 2018. Cows were fed forage and concentrates. Milk samples were immediately added with Azidiol preservative (Nova Chimica Srl, Italy) and analysed with MilkoScan^TM^ 7 (Foss Electric A/S, Hillerød, Denmark) for milk composition in the laboratory of the South Tyrolean Dairy Association (Bolzano, Italy). Mid-infrared spectra of the analysed samples were also available. Starting spectra comprised 1060 data points between 5012 and 686 cm^−1^. An aliquot of 10 mL of samples was stored at 4 °C and the protocol for diffusible and micellar minerals separation was applied within 12 h as described by Franzoi et al. (2018) [12]. Briefly, 10 mL of milk was warmed at 38 °C for 2 h in order to reach mineral equilibrium between diffusible and micellar phases, and then calf rennet (1:3000 wt/wt) was added. Milk was incubated at 36 °C for 30 min; the curd was then cut and re-incubated for 30 min. After centrifugation, 5 mL of whey was collected, and 5 mL of ultrapure water was added to the samples and left to equilibrate for 1 h after mixing. Finally, samples were centrifuged and 5 mL of diluted whey was collected.

Mineral extraction from starting milk, whey and diluted whey was carried out through acid mineralisation, whereas mineral quantification was obtained through inductively coupled plasma optical emission spectrometry. Finally, diffusible and micellar mineral fractions were calculated as proposed by Franzoi et al. (2018) [12].

### 2.2. Prediction Models

The normal distribution of traits in the calibration dataset was checked using the Shapiro-Wilk test. Outliers for reference measures were defined as the values deviating more than 3 standard deviations from the mean of each trait. Spectral variables were transformed to absorbance by applying log_10_ of reciprocal of the transmittance. Spectral regions to develop prediction models were selected as the “good spectrum” wavelengths according to the infrared instrument manufacturer. The calibration dataset was checked for spectral outliers using Mahalanobis distance and no outliers were detected. The final dataset comprised 450 spectral variables in the intervals 964.5 to 1562.5 cm^−1^, 1720.7 to 2291.7 cm^−1^ and 2415.1 to 2970.7 cm^−1^, from 91 samples.

Prediction models and fitting statistics were obtained using SAS 9.4 (SAS Institute Inc., Cary, NC, USA). Backward interval partial least squares analysis was performed according to Zou et al. (2007) [23]. Spectra were divided into 45 intervals, including 10 variables in each interval, and PROC PLS of SAS 9.4 was iteratively performed excluding one interval at a time. Predicted residual error sum of squares (PRESS) was calculated for each iteration. The interval to be excluded from the subsequent BiPLS round was the one resulting in the lowest PRESS statistic when left out. The procedure was iterated until only one interval remained [22]. For each iteration, the number of latent variables (LV) to perform partial least squares (PLS) procedure was defined as the minimum number of LV from 1 to 10 to achieve the lowest PRESS, with *p* > 0.10. Root mean square error in leave-one-out cross validation (RMSE_CV_) was calculated for each BiPLS round and the model with the best performance was selected as the final prediction model. For comparison, PLS was performed using the same parameters as the BiPLS analysis, including all spectra wavenumbers to develop the prediction models. Fitting statistics of PLS and BiPLS models were the coefficient of determination in cross-validation (R^2^_CV_) and the ratio of performance to deviation in cross validation (RPD_CV_), calculated as the ratio between SD and RMSE_CV_. Moreover, the difference between R^2^_CV_ of BiPLS and PLS models (ΔR^2^_CV_) for each mineral was calculated.

## 3. Results and Discussion

Fat, protein, lactose and pH averaged 4.03%, 3.47%, 4.75% and 6.65%, respectively (Table 1). Regarding milk mineral composition, total Ca averaged 123.26 mg/100 mL, with micellar Ca being about 3.5-fold the diffusible Ca. Total P averaged 102.03 mg/100 mL, with micellar P being nearly twofold the diffusible P. Potassium was primarily present more in the diffusible (121.38 mg/100 mL) than in the micellar phase (26.12 mg/100 mL). On the other hand, Mg was almost evenly distributed between micellar and diffusible phases (58% and 42%, respectively). Finally, about 99% of Na was in the diffusible phase, with a total amount that averaged 36.63 mg/100 mL. Overall, the concentration of minerals in the diffusible phase was lower than that reported by Gaucheron (2005) [22]. Such results can be attributed to the peculiar quantification method applied in the present study. Indeed, the proposed quantification does not suffer the bias due to the difference between the correction factor for the excluded volume, applied equally to all the samples to correct for the diffusible minerals trapped in the curd, and the real excluded volume, influenced by milk solids content.

**Figure 1:**
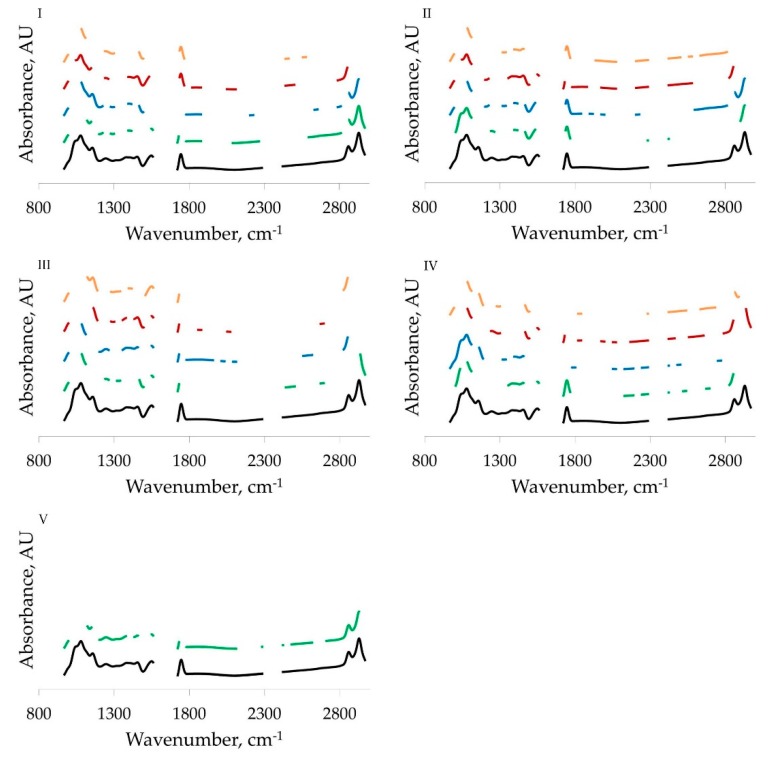
Spectral regions selected using backward interval partial least squares analysis. (**I**) Ca, (**II**) P, (**III**) K, (**IV**) Mg, (**V**) Na. (Black) full spectrum (mean); (green) total mineral (mg/100 mL); (blue) micellar mineral (mg/100 mL); (red) diffusible mineral (mg/100 mL); (orange) ratio between micellar and diffusible fractions (*w*/*w*). Absorbance is given in arbitrary units (AU).

Spectral regions selected through BiPLS analysis are depicted in Figure 1. Spectral regions for diffusible and micellar minerals are not complementary when compared with total minerals, suggesting that the overlapped spectral regions are likely correlated with both micellar and diffusible minerals. All calibrations included at least one interval from the spectral region related to C=O and N–H absorption of proteins (1700 to 1500 cm^−1^). Most of the calibrations included also intervals around 1100 cm^−1^, related to carbohydrates C–O and C–C stretching, and wavenumbers around 1400 cm^−1^ and between 2770 and 2980 cm^−1^, related to lipids. This could be related to the effect of minerals on shifting signals of milk components, e.g., Ca interaction with side chains of casein amino acids, and the correlations of total, micellar and diffusible minerals with the protein, fat or carbohydrate content of milk [19,24].

In general, BiPLS enhanced the accuracy of prediction models compared with PLS analysis, with a ΔR^2^_CV_ that ranged from 0.07 (micellar K) to 0.25 (total P; Table 2). Fitting statistics revealed moderate to low predictive performance for the analysed traits. Considering the total content of each element, R^2^_CV_ and RPD_CV_ of prediction models developed using BiPLS analysis ranged from 0.55 to 0.87 and from 1.49 to 2.73 for total K and total P, respectively. Prediction performances of diffusible minerals were similar to those of total minerals, with an RPD_CV_ that ranged from 1.52 (diffusible Mg) to 2.09 (diffusible Ca). In general, prediction of micellar minerals showed fitness statistics slightly poorer than those of total and diffusible minerals, except for micellar K, whose prediction was particularly difficult, probably for the small fraction of this mineral present in the micellar phase (18%) and the relatively high uncertainty of the quantification method to determine micellar K [12]. Considering the strong relationship between the amount of total minerals and the amount of minerals in each fraction [25], the predictive ability of MIRS was also tested for mineral partitioning in the phases, expressed as *w*/*w*. Prediction models effectively predicted mineral partitioning, even if with lower accuracy than the absolute amount.

Results for mid-infrared predictions of total milk minerals were in agreement with findings reported in the literature [18,21,26]. Taking into account micellar and diffusible minerals, the results of the present study indicated better prediction performances compared with Malacarne et al. (2018) [17]; such results may be related to the different origin of collected samples (bulk vs. individual), the different method for reference data collection and the use of BiPLS instead of simple PLS analysis as the method to develop prediction models.

## 4. Conclusions

The present study reported the ability of MIRS models to predict micellar and diffusible major minerals in milk. Backward interval partial least squares analysis effectively improved prediction models compared with PLS analysis. Moderate prediction performances were achieved for Ca, P and Mg, and poor predictive statistics were achieved for K, in particular in the micellar fraction. Sodium predictions in the two phases were not possible, given that more than 99% of Na is present in the diffusible phase. Future research will focus on the use of the developed equations to predict mineral fractions on a large scale and will consider the influence of milk mineral partition on cheese-making properties.

## Figures and Tables

**Table 1:** Descriptive statistics of milk composition, milk minerals (mg/L) and ratio between micellar and diffusible fractions (*w*/*w*).

Trait		Mean	SD	CV, %	Minimum	Maximum
Milk composition, %						
Fat		4.03	0.63	15.63	2.68	5.84
Protein		3.47	0.34	9.80	2.54	4.28
Lactose		4.75	0.21	4.42	4.10	5.15
pH		6.65	0.06	0.90	6.49	6.79
Minerals						
Ca	Total, mg/100 mL	122.07	10.29	8.43	92.42	146.68
	Diffusible, mg/100 mL	27.95	5.63	20.13	19.00	49.33
	Micellar, mg/100 mL	94.12	10.81	11.48	66.96	123.41
	Micellar/Diffusible, *w*/*w*	3.51	0.84	24.03	1.57	5.93
P	Total, mg/100 mL	99.86	9.03	9.04	73.47	119.20
	Diffusible, mg/100 mL	36.51	4.86	13.30	24.28	48.90
	Micellar, mg/100 mL	63.35	8.98	14.18	41.85	84.39
	Micellar/Diffusible, *w*/*w*	1.77	0.40	22.35	1.14	2.67
K	Total, mg/100 mL	147.56	10.63	7.21	125.07	174.25
	Diffusible, mg/100 mL	120.94	10.60	8.77	97.43	147.14
	Micellar, mg/100 mL	26.62	9.42	35.40	4.72	52.33
	Micellar/Diffusible, *w*/*w*	0.22	0.09	39.46	0.04	0.49
Mg	Total, mg/100 mL	10.99	1.12	10.23	8.79	14.06
	Diffusible, mg/100 mL	6.38	0.82	12.90	4.44	8.51
	Micellar, mg/100 mL	4.62	0.94	20.30	2.53	7.01
	Micellar/Diffusible, *w*/*w*	0.74	0.19	25.70	0.34	1.40
Na	Total, mg/100 mL	37.55	7.94	21.15	27.73	65.71

Na was not found in micellar phase and thus it is presented as total Na only. Abbreviations are as follows: SD, standard deviation; CV, coefficient of variation.

**Table 2:** Fitting statistics of prediction models for milk minerals and ratio between micellar and diffusible fractions (*w*/*w*) in cross-validation obtained using partial least squares (PLS) and backward interval PLS (BiPLS) analysis (n = 91).

Trait		PLS				BiPLS					ΔR^2^_CV_
		LV	RMSE_CV_	R^2^_CV_	RPD_CV_	NV	LV	RMSE_CV_	R^2^_CV_	RPD_CV_	
Ca	Total, mg/100 mL	9	5.86	0.68	1.76	230	10	4.77	0.79	2.16	0.11
	Diffusible, mg/100 mL	5	3.15	0.69	1.79	180	9	2.69	0.77	2.09	0.08
	Micellar, mg/100 mL	8	6.35	0.66	1.70	160	10	5.30	0.76	2.04	0.10
	Micellar/Diffusible, *w*/*w*	5	0.58	0.52	1.45	90	6	0.36	0.69	1.78	0.17
P	Total, mg/100 mL	5	5.58	0.62	1.62	100	10	3.30	0.87	2.73	0.25
	Diffusible, mg/100 mL	7	2.91	0.64	1.67	230	10	2.56	0.73	1.90	0.09
	Micellar, mg/100 mL	5	6.27	0.52	1.43	210	10	4.76	0.73	1.89	0.21
	Micellar/Diffusible, *w*/*w*	7	0.27	0.56	1.49	220	10	0.22	0.68	1.77	0.12
K	Total, mg/100 mL	10	8.58	0.35	1.24	100	10	7.14	0.55	1.49	0.20
	Diffusible, mg/100 mL	6	7.58	0.49	1.40	100	9	6.92	0.58	1.53	0.09
	Micellar, mg/100 mL	7	8.69	0.18	1.08	180	7	8.19	0.25	1.15	0.07
	Micellar/Diffusible, *w*/*w*	3	0.09	0.09	1.04	90	7	0.08	0.29	1.17	0.20
Mg	Total, mg/100 mL	7	0.74	0.57	1.52	140	10	0.64	0.68	1.76	0.11
	Diffusible, mg/100 mL	5	0.62	0.43	1.32	190	7	0.54	0.57	1.52	0.14
	Micellar, mg/100 mL	5	0.68	0.48	1.38	160	7	0.53	0.68	1.77	0.20
	Micellar/Diffusible, *w*/*w*	4	0.16	0.33	1.22	140	6	0.13	0.56	1.51	0.23
Na	Total, mg/100 mL	8	4.85	0.63	1.64	290	10	4.02	0.75	1.98	0.12

Na was not found in micellar phase and thus it is presented as total Na only. Abbreviations are as follows: LV, number of latent variables for calibration; RMSE_CV_, root mean square error in cross-validation; R^2^_CV_, coefficient of determination in cross-validation; RPD_CV_, ratio of performance to deviation in cross-validation; NV, number of spectral variables selected in the model; ΔR^2^_CV_, difference between R^2^_CV_ of BiPLS and PLS.

## References

[1] Lombardi-Boccia G., Aguzzi A., Cappelloni M., Lullo G.D., Lucarini M. (2003). Total-diet study: Dietary intakes of macro elements and trace elements in Italy. Brit. J. Nutr..

[2] Lucey J.A., Fox P.F. (1993). Importance of calcium and phosphate in cheese manufacture: A review. J. Dairy Sci..

[3] Malacarne M., Franceschi P., Formaggioni P., Sandri S., Mariani P., Summer A. (2014). Influence of micellar calcium and phosphorus on rennet coagulation properties of cows milk. J. Dairy Res..

[4] De la Fuente M.A. (1998). Changes in the mineral balance of milk submitted to technological treatments. Trends Food Sci. Tech..

[5] Holt C. (2004). An equilibrium thermodynamic model of the sequestration of calcium phosphate by casein micelles and its application to the calculation of the partition of salts in milk. Eur. Biophys. J..

[6] Gaucheron F. (2013). Milk Minerals, Trace Elements, and Macroelements. Milk and Dairy Products in Human Nutrition.

[7] Gaucheron F. (2011). Milk and dairy products: A unique micronutrient combination. J. Am. Coll. Nutr..

[8] Holt C., Carver J.A., Ecroyd H., Thorn D.C. (2013). Invited review: Caseins and the casein micelle: Their biological functions, structures, and behavior in foods. J. Dairy Sci..

[9] Manuelian C.L., Penasa M., Visentin G., Zidi A., Cassandro M., De Marchi M. (2018). Mineral composition of cow milk from multibreed herds. Anim. Sci. J..

[10] Chen B., Lewis M.J., Grandison A.S. (2014). Effect of seasonal variation on the composition and properties of raw milk destined for processing in the UK. J. Food Chem..

[11] De la Fuente M.A., Fontecha J., Juárez M. (1996). Partition of main and trace minerals in milk: Effect of ultracentrifugation, rennet coagulation, and dialysis on soluble phase separation. J. Agric. Food Chem..

[12] Franzoi M., Niero G., Penasa M., Cassandro M., De Marchi M. (2018). Technical note: Development and validation of a new method for the quantification of soluble and micellar calcium, magnesium, and potassium in milk. J. Dairy Sci..

[13] deMan J.M. (1962). Measurement of the partition of some milk constituents between the dissolved and colloidal phases. J. Dairy Res..

[14] Khan N., Choi J.Y., Nho E.Y., Hwang I.M., Habte G., Khan M.A., Park K.S., Kim K.S. (2014). Determination of mineral elements in milk products by inductively coupled plasma-optical emission spectrometry. Anal. Lett..

[15] De Marchi M., Toffanin V., Cassandro M., Penasa M. (2014). Invited review: Mid-infrared spectroscopy as phenotyping tool for milk traits. J. Dairy Sci..

[16] McDermott A., Visentin G., De Marchi M., Berry D.P., Fenelon M.A., O’Connor P.M., Kenny O.A., McParland S. (2016). Prediction of individual milk proteins including free amino acids in bovine milk using mid-infrared spectroscopy and their correlations with milk processing characteristics. J. Dairy Sci..

[17] Malacarne M., Visentin G., Summer A., Cassandro M., Penasa M., Bolzoni G., Zanardi G., De Marchi M. (2018). Investigation on the effectiveness of mid-infrared spectroscopy to predict detailed mineral composition of bulk milk. J. Dairy Res..

[18] Soyeurt H., Bruwier D., Romnee J.-M., Gengler N., Bertozzi C., Veselko D., Dardenne P. (2009). Potential estimation of major mineral contents in cow milk using mid-infrared spectrometry. J. Dairy Sci..

[19] Visentin G., McDermott A., McParland S., Berry D.P., Kenny O.A., Brodkorb A., Fenelon M.A., De Marchi M. (2015). Prediction of bovine milk technological traits from mid-infrared spectroscopy analysis in dairy cows. J. Dairy Sci..

[20] Visentin G., Penasa M., Niero G., Cassandro M., De Marchi M. (2018). Phenotypic characterisation of major mineral composition predicted by mid-infrared spectroscopy in cow milk. Ital. J. Anim. Sci..

[21] Visentin G., Penasa M., Gottardo P., Cassandro M., De Marchi M. (2016). Predictive ability of mid-infrared spectroscopy for major mineral composition and coagulation traits of bovine milk by using the uninformative variable selection algorithm. J. Dairy Sci..

[22] Xiaobo Z., Jiewen Z., Povey M.J.W., Holmes M., Hanpin M. (2010). Variables selection methods in near-infrared spectroscopy. Anal. Chim. Acta.

[23] Zou X., Zhao J., Li Y. (2007). Selection of the efficient wavelength regions in FT-NIR spectroscopy for determination of SSC of ‘Fuji’ apple based on BiPLS and FiPLS models. Vib. Spectrosc..

[24] Dufour É. (2009). Principles of Infrared Spectroscopy. Infrared Spectroscopy for Food Quality Analysis and Control.

[25] Gaucheron F. (2005). The minerals of milk. Reprod. Nutr. Develop..

[26] Toffanin V., De Marchi M., Lopez-Villalobos N., Cassandro M. (2015). Effectiveness of mid-infrared spectroscopy for prediction of the contents of calcium and phosphorus, and titratable acidity of milk and their relationship with milk quality and coagulation properties. Int. Dairy J..

